# Hypnosis support in anaesthesia is rarely used in German anaesthesia departments - a nationwide survey among leading physicians of anaesthesia departments

**DOI:** 10.1186/s12871-024-02705-4

**Published:** 2024-09-06

**Authors:** Lisa Bügers, Anna Wähner, Ann-Kristin Schubert, Hanns-Christian Dinges, Alexander Torossian, Christian Volberg

**Affiliations:** 1 Department of Anaesthesiology and Intensive Care Medicine, University Hospital Marburg, Philipps University of Marburg, Baldingerstraße, 35043 Marburg, Germany; 2 Faculty of Medicine, Research Group Medical Ethics, Philipps University of Marburg, Marburg, Germany

**Keywords:** Anaesthesia, Hypnosis, Hypnotherapy, Progressive muscle relaxation, Relaxation techniques

## Abstract

**Background:**

The aim of this study was to investigate whether and to what extent perioperative hypnosis and relaxation techniques are used in German anaesthesia departments, what they are, where any difficulties in their application lie and how great the interest in this type of therapy is. Another research question was to find out whether there are specialist areas in which these methods are used more frequently than in other specialist areas.

**Methods:**

A descriptive survey was conducted by means of a questionnaire in all hospitals with anaesthesia departments in Germany. 1124 questionnaires were sent out by post. The survey period was five months from 27/02/2023 to 31/07/2023. The clinic directors of all anaesthesiology departments in German hospitals were surveyed.

**Results:**

476 departments (42%) responded by pre-paid envelope. Of these, only 39 (8%) use hypnosis and relaxation techniques perioperatively. These are mostly progressive muscle relaxation, hypnotic trance according to Erickson, calming words and suggestions or the use of virtual reality (e.g. using VR-glasses).

**Conclusions:**

Hypnosis techniques have been shown in many studies to be effective in increasing patient comfort (less anxiety and stress) and reducing both the need for medication and perioperative pain. The therapy is rarely used in Germany, although once established it can be easily integrated into perioperative procedures. Many departments have shown great interest in the topic. In the interests of patients, a structural change should be considered to promote the use of hypnotic procedures.

## Background

Hypnotherapy has been recognized in Germany as a scientifically based method by the *Scientific Advisory Board for Psychotherapy* since 2006. It is mainly used in the areas of addiction disorders and psychological and social factors in somatic illnesses [1]. In Germany, there are various training courses for medical and psychotherapeutic staff, which are mainly organized and certified by scientifically based German hypnosis associations such as the Milton Erickson Society (MEG), the German Society for Hypnosis and Hypnotherapy (DGH) and the German Society for Dental Hypnosis (DGZH).

Nevertheless, neither hypnosis nor hypnotherapy are protected terms in Germany. As a result, there are many unscientific offers and training courses and there seem to be some prejudices [2].

Hypnosis is a form of therapy that draws on the patient’s own resources and attempts to utilize them in a state of trance. The focus is placed on certain ideas and thoughts so that the perception of everyday external stimuli and feelings is eliminated during the trance state. As a result, patients experience a kind of change in consciousness. Experiences and perspectives can be reconnected and thus changed [3, 4].

Progressive muscle relaxation (PMR) according to Jacobson is a well-known method. Originally developed by Dr. Edmund Jacobson in the 1920s, this time-consuming form of therapy has been further developed and modified over the years. The aim of these time-saving variants was and is to achieve sufficiently deep relaxation more quickly [5]. In addition, these modified methods have been better scientifically analysed and thus evidence of their effectiveness could be found [6]. A further advantage is that patients who have learnt the procedure once can use it independently and are therefore less dependent on the presence of a therapist.

Hypnotherapy, according to Milton Erickson, is a form of therapy in which communication is used to put patients into a state of hypnotic trance. The procedure can be individually tailored to the person and is mainly based on verbal images and the patient’s imagination. The aim is to utilize the trance that has occurred and the patient’s heightened awareness to bring about mental or physical changes, such as increased relaxation. Erickson referred to this process as “Utilization”, i.e. the harnessing of the patient’s own resources [7].

Another and more modern form of hypnosis is the use of virtual reality (VR), for example with VR glasses. These goggles show pre-programmed environments that are generally perceived as beautiful and peaceful to maintain a trance-like or dissociative state in the patient even more effectively. Hypnosis can also be induced using these goggles and auditory suggestions. Users are also less reliant on specially trained hypnotherapists, which could make it easier to use. There is also another form that combines the use of hypnosis and VR which is called Virtual Reality Hypnosis (VRH). This combination has been shown to be particularly effective in terms of pain intensity and pain-related discomfort [8].

In anaesthesia, hypnosis techniques can have many positive effects on patients. For example, it can bring about an improvement in an acute pain situation [9]. The need for hypnotic medication can also be reduced under certain circumstances [10, 11]. Both have a particularly favourable effect on patients in risk groups. In groups of patients who regularly consume drugs and therefore have higher doses of medication and thus an increased risk of drug overdose, it has been shown that hypnotherapy improves the response to a lower dose of medication during dental treatment [12]. In 2007, Montgomery et al. were able to prove in a randomized controlled study that 15 min of pre-operative hypnosis leads to lower propofol and lidocaine requirements during general anaesthesia and to fewer side effects (pain, nausea, fatigue). However, it did not lead to a significantly lower consumption of fentanyl or midazolam [13]. Due to the reduced need for medication, side effects and overall costs can be reduced and a shorter post-operative phase can be observed [10, 14]. In some patient groups, general anaesthesia can even be dispensed with completely [15, 16]. This also applies to surgical procedures and dental patients [17].

Particularly in geriatric patients, surgery under general anaesthesia is associated with the risk of post-operative delirium or other peri- and post-operative complications [18, 19]. Regional and local anaesthesia procedures are therefore suitable for this group, although they are associated with an increased cortisol level as a marker for increased stress [20]. It would therefore make sense to discover procedures that lead to a reduction in stress in patients by other means [21]. It has been shown that parasympathetic effects can be demonstrated in patients treated with hypnosis procedures [22]. Courtois-Amiot et al. were able to illustrate in a randomized controlled study in 2022 that patients with Alzheimer’s disease or cognitive impairment also benefit from suggestive hypnosis therapy before diagnostic lumbar punctures. Most of them stated after the procedure that the experience was better than expected [23].

An improved postoperative outcome in terms of faster recovery after surgery and thus faster discharge from hospital was also demonstrated through the use of hypnosis procedures [24, 25]. In 2017, Zorilla-Vaca et al. showed that this also applies to neurosurgical procedures under regional anaesthesia [26]. Anxiety-reducing and drug-saving effects have also been demonstrated in children, both in dentistry and for minor diagnostic procedures [27–30].

But it is not just patients who benefit from hypnosis. Boselli et al. showed in 2012 that hypnosis practitioners with professional training have a reduced risk of burnout [31].

As there is currently no data available on the perioperative use of hypnosis and relaxation techniques in Germany, or the training programs offered in hospitals, our research group conducted a nationwide survey on this topic. Better knowledge about the use of hypnosis methods in Germany could provide starting points for further establishing hypnotherapy or for increasing the number of training programs.

## Methods

After an extensive literature review, a 26-question questionnaire was developed by the study team, reviewed by two independent colleagues, and then adapted for comprehensibility by five independent medical colleagues. The questionnaire is divided into two sections. The general first section contains questions on the demographic details of the participating clinic and ends with a key question on the use of hypnosis techniques. If this question is answered with “yes”, specific questions on the use of hypnosis and relaxation techniques in the perioperative process follow. If the key question is answered with “no”, this is followed by a few questions on the general evaluation of hypnosis and interest in further training in this area.

### Ethical approval

for this study (Ethical Committee N° 22–177 ANZ) was provided by the institutional review board of Philipps-University Marburg, Germany (Chairperson Prof C. Seifart) on 13 January 2023. After positive vote, the questionnaire was sent by mail on 27/02/23 as a paper version to all anaesthesia departments in German hospitals (1124 in total) and addressed to the respective medical director of the anaesthesia department. The addresses were acquired from the German hospital register [32]. The questionnaire was accompanied by a cover letter with information about the background to the survey and a pre-paid return envelope. Within the cover letter, the head of the respective anaesthesia department was asked to participate in the anonymized survey and to complete the questionnaire according to the circumstances in the department under his or her management.

Microsoft Excel^®^ Version 16.6 was used for the descriptive data analysis and presentation of the data is based on the SQUIRE 2.0 guidelines [33].

## Results

### General information

A total of 476 completed questionnaires (42%) were returned in the period from 27/02/23 to 31/07/23, of which 474 questionnaires were included in the analysis. Two questionnaires were not analysed as more than half of the questions were not answered. Ten questionnaires could not be delivered and were therefore omitted from the statistical analysis.

Most participating hospitals were primary and standard care clinics (52%), treating between 1000 and 5000 patients per year (37%) and had up to 25 medical staff in the anaesthesia department (60%). Most of the participating clinics treated both children and adults (81%). Further demographic data can be found in Table 1.

**Table 1:** Demographic information of the participating clinics

	Total	%
**Which category does your hospital correspond to? (** ***n*** ** = 473)**		
Basic and standard care providers	245	51,8
Specialist hospital (e.g. orthopaedic clinic)	130	27,5
Tertiary care provider	37	7,8
University Hospital	26	5,5
Others	40	8,5
**How many medical staff do you have in your anaesthesia department? (** ***n*** ** = 472)**		
1–25	285	60,4
26–50	110	23,3
51–100	52	11
> 100	25	5,3
**On average**, **how many anaesthesiologic patients do you care for in your clinic each year? (*n* = 472)**		
< 1000	14	3
1000–5000	175	37,1
5000-10.000	150	31,8
> 10.000	133	28,2
**Do you look after children and/or adults? (** ***n*** ** = 472)**		
Only children	3	0,6
Only adults	86	18,2
Children and adults	383	81,1
**Are hypnosis or relaxation procedures used perioperatively in your clinic? (** ***n*** ** = 473)**		
Yes	39	8,3
No	434	91,7

### Use of hypnosis and relaxation techniques

Of the 474 departments that responded to the questionnaires, 39 (8%) use hypnosis and/or relaxation techniques perioperatively. The remaining 435 departments stated that they did not use any such procedures. Hypnosis and relaxation techniques were used by 60% each in combination with local/regional anaesthesia, general anaesthesia or (analgo-) sedation and following the individual decision of the anaesthesiologist.

**Fig. 1:**
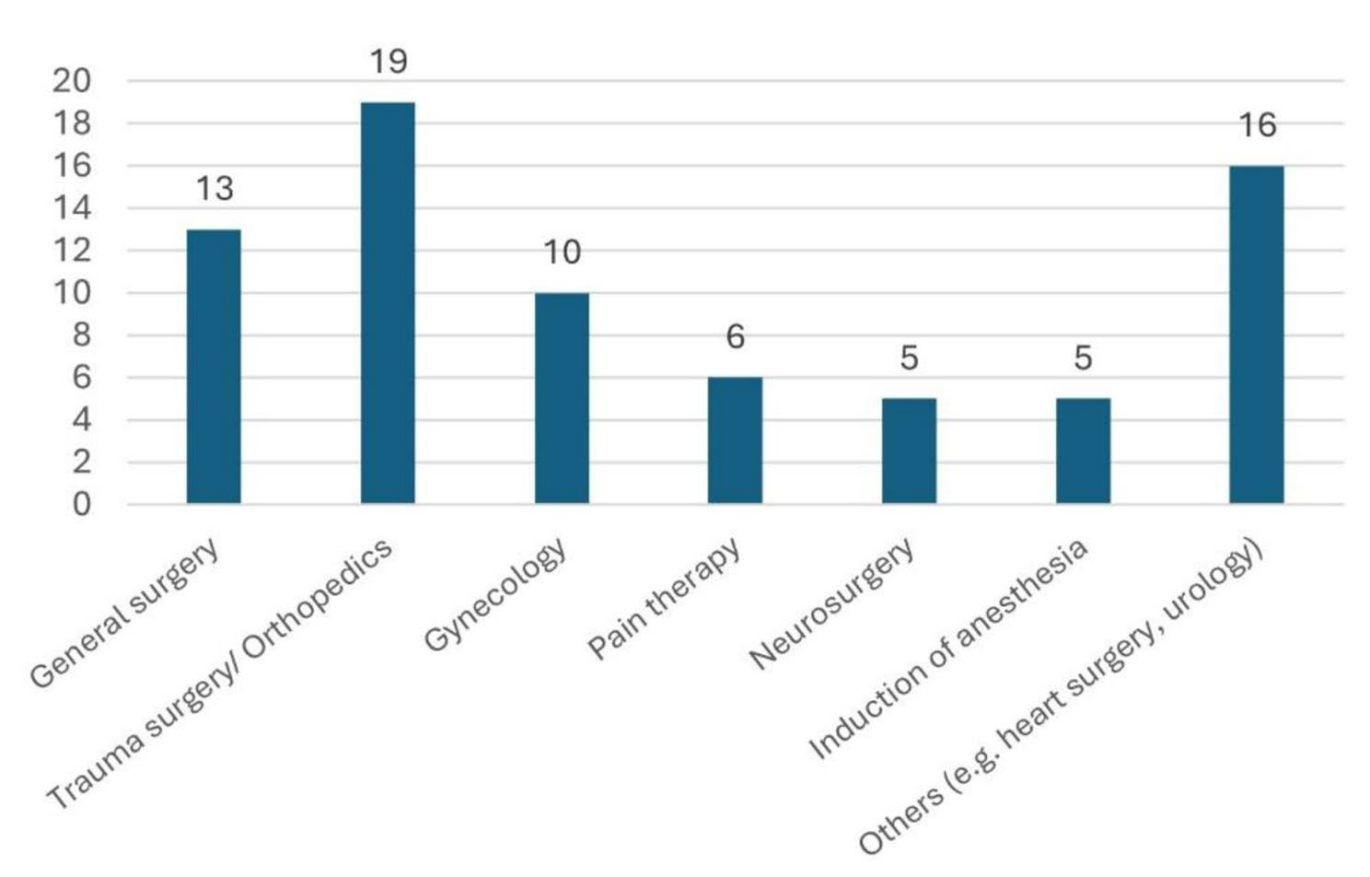
Specialities in which hypnosis is most commonly used (triple selection)

The main areas of application in hospitals were perioperatively in operating theatres with inpatient care (77%), ambulatory surgery (28%) and pain therapy (51%). Further differentiation within this data by specialty revealed that hypnosis and relaxation techniques were mostly used in orthopaedics/trauma surgery (26%), in general surgery (18%) and in gynaecological surgery (14%) (see Fig. 1).

**Fig. 2:**
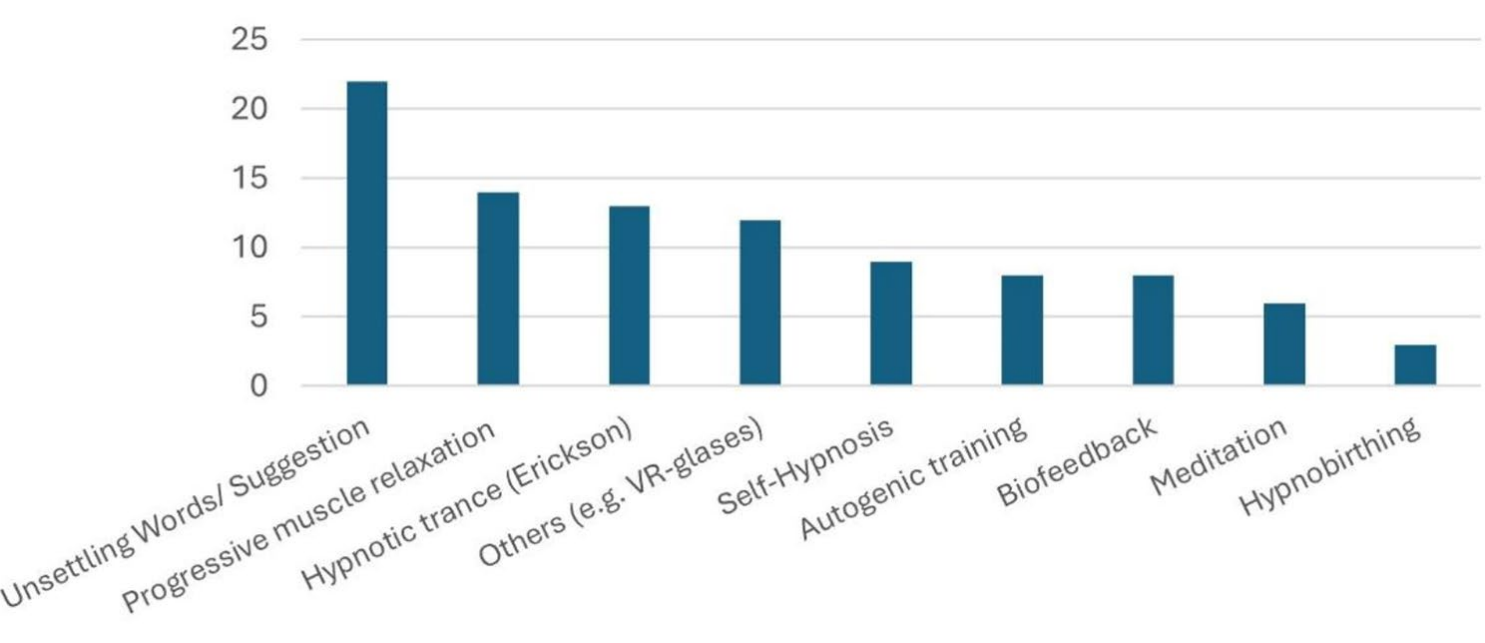
Most commonly used treatments (multiple choice)

In pain therapy, the methods of progressive muscle relaxation according to Jacobson (65%), hypnotic trance according to Erickson (55%) and autogenic training (40%) were used in particular (see Fig. 2). On average, around 64 (maximum 200, minimum 1–2) patients per hospital were treated with hypnosis per month, mostly (78%) with a single therapy session.

Progressive muscle relaxation according to Jacobson (37%) and hypnotic trance according to Erickson (34%) were also used outside of pain therapy, in the context of anaesthesia. In addition, very often (58%) calming words or suggestions via headphones were used during anaesthesia. Modern technical procedures such as VR glasses were used in 11% of the responding institutions.

### Training and further education/further interest

In most hospitals (85%), hypnosis techniques were performed by specially trained staff. These certified training courses were most frequently obtained from the Milton Erickson Society (MEG), followed by medical hypnosis courses, trained psychotherapists, in-house training and hypnotherapists. According to our survey, there was an average of 4–5 trained employees per department. However, the highest number (mentioned once) was 31, while the most frequently mentioned number was one person.

About half (49%) of the departments that used hypnosis techniques also offer training in hypnosis. Of the departments that did not use hypnosis techniques, 92% did not offer any training on this topic.

**Fig. 3:**
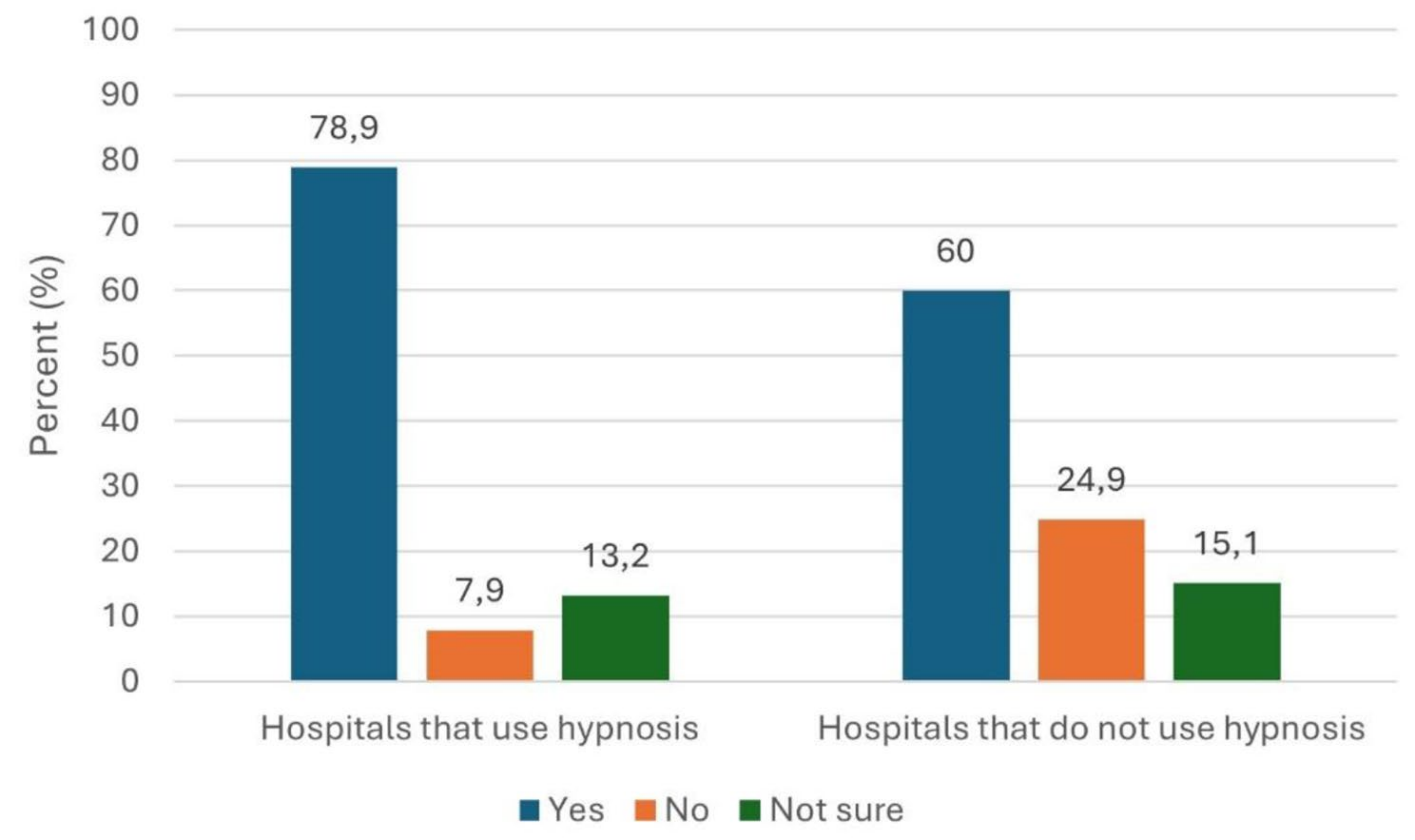
Interest in learning more about hypnosis

Overall, 62% of clinic directors expressed a general interest in more information about hypnosis and relaxation techniques. Among those who did not use hypnosis, the figure was 60%. Notably, 79% of hospitals using hypnosis were also interested in further training (compare Fig. 3).

**Fig. 4:**
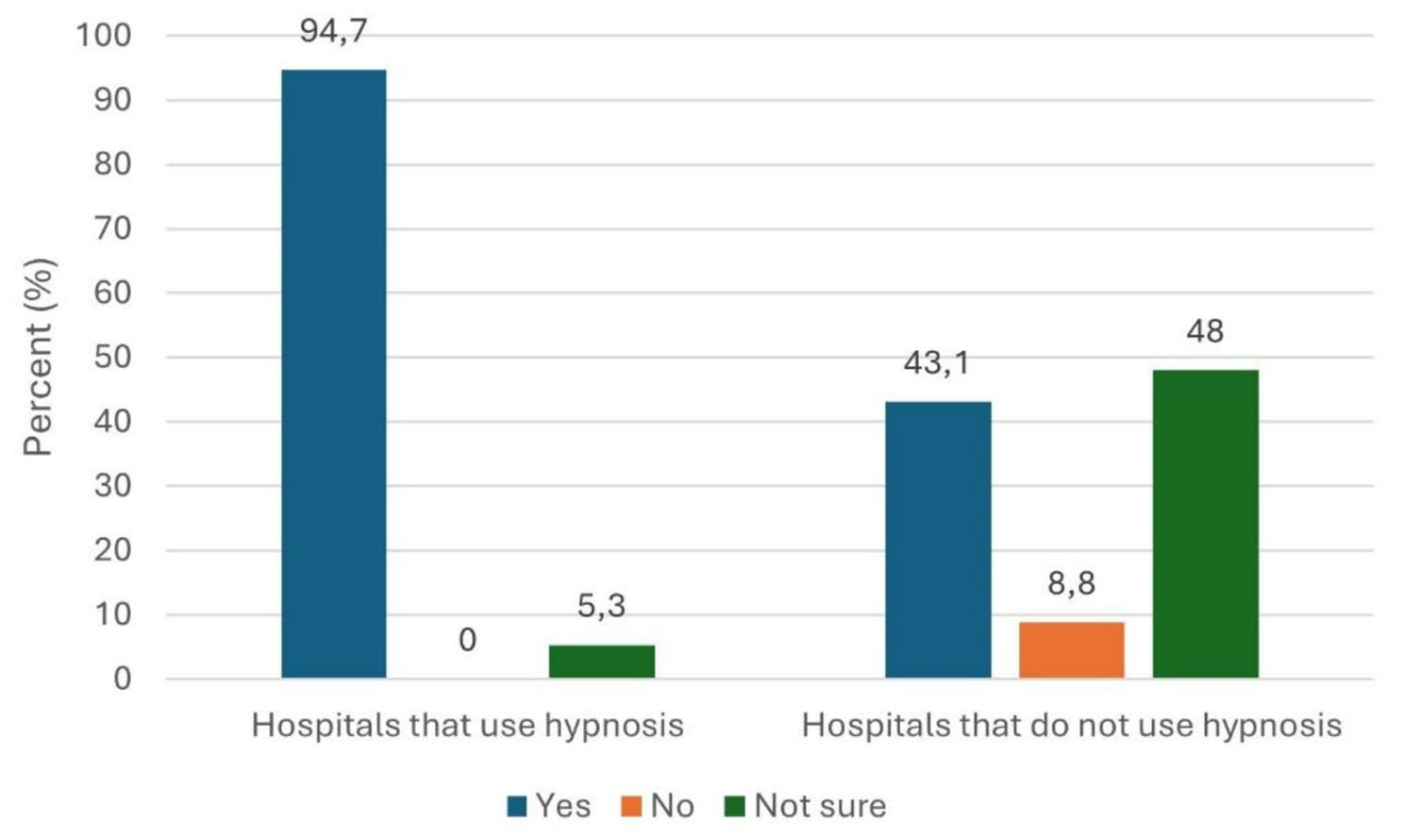
Are hypnosis procedures considered useful?

47% of the hospitals/anaesthesia departments considered the use of hypnosis procedures to be useful, regardless of whether they actually used hypnosis procedures themselves or not. 43% of hospitals that did not use hypnosis still considered its use useful, while 53% could imagine using hypnosis (see Fig. 4).

### Application goals

**Fig. 5:**
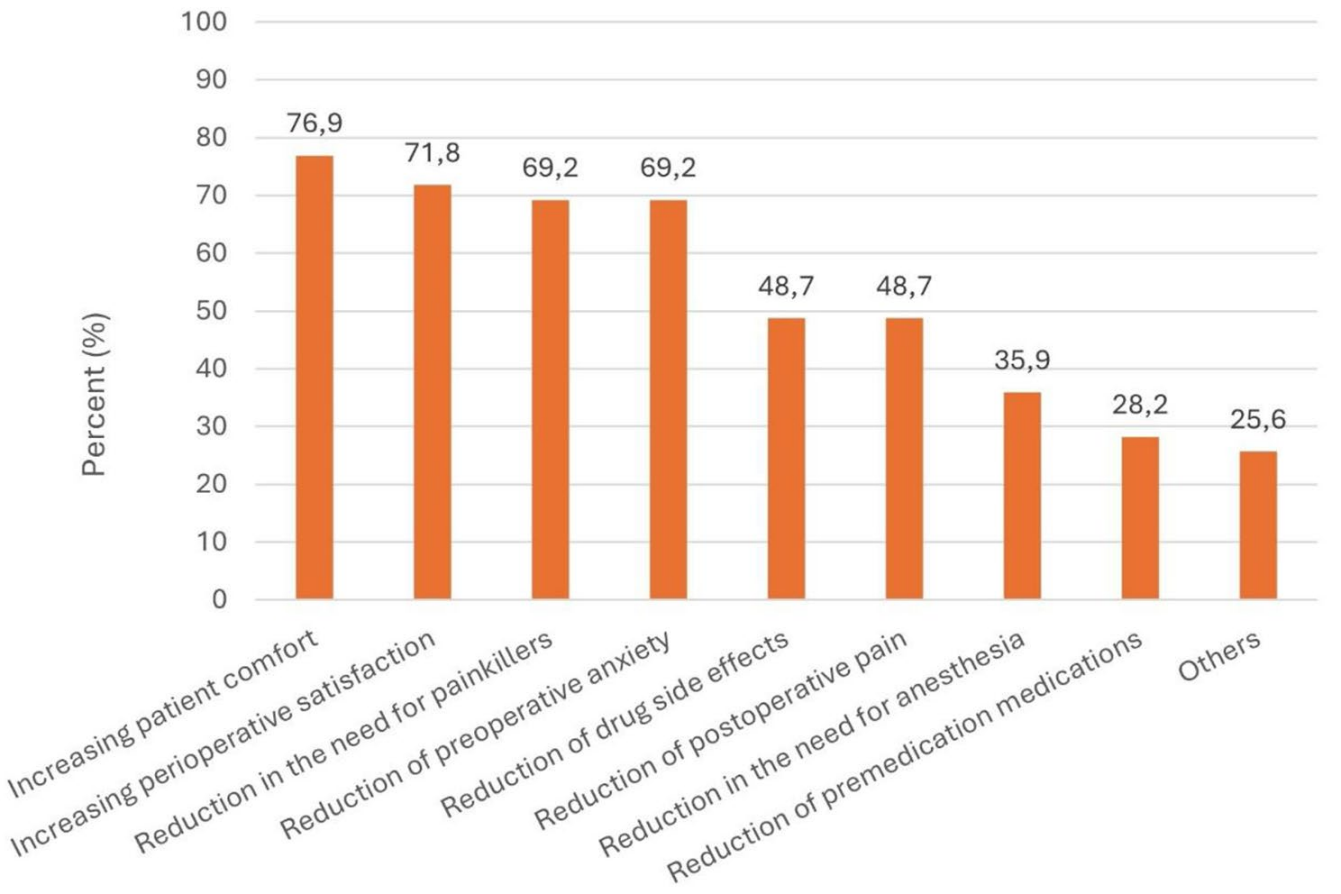
Therapeutic goals for the use of hypnosis (multiple choice)

Departments cited improving patient comfort, perioperative satisfaction and reducing the need for pain medication, medication side effects and preoperative anxiety as the main objectives (compare Fig. 5).

### Application difficulties

According to the survey, hypnotic procedures could be integrated well or very well (36% in each case) into perioperative procedures in hospitals using them. However, problems were reported, the most common being staff (47%) and time difficulties (56%). Lack of information about the topic was also seen as a problem by 32%.

## Discussion

Most anaesthesia departments do not offer or use hypnosis or relaxation techniques. According to our survey, only 8% of the responding departments use such forms of therapy. It is likely that there are even fewer, as only 42% of the questionnaires were answered and could be included in the calculation. The question therefore arises as to why hypnotherapy seems to play a minor role in German anaesthesiology.

One possible reason could be that anaesthesia staff are not aware of the possibilities of hypnosis and the positive effects described above. In this context, an Italian study, which surveyed 78 nurses on this topic, concluded that the reasons for the low use of hypnosis procedures were to be found in social prejudices and the lack of theoretical training during their studies [34]. In Germany, too, hypnosis is neither included in the *licensing regulations for doctors* nor in the *training and examination regulations for the nursing professions* [35, 36]. A review by Iserson from 2014 shows that hypnosis methods are little used overall, even though they are safe, quick and inexpensive. This may be due to insufficiently proven benefits and a lack of training and education [37].

Following an enquiry to the Milton Erickson Society (MEG) at the end of September 2023, we found out that there are currently only around 2,500 hypnotherapists in Germany who have been systematically trained and certified by the MEG, with around 70 to 90 new hypnotherapists every year. This fact could explain the personnel problem in the performance of perioperative hypnosis. The MEG trains both medical and non-medical staff. This low rate is also reflected in the surveyed anaesthesia departments.

The low number of in-hospital training courses and the high level of interest in more information may also explain the staffing problem. Many of the German clinic directors consider the use of hypnosis and relaxation methods to be useful, suggesting that it is not a fundamental aversion to the subject that is the reason for the low level of use of hypnosis and relaxation methods in Germany. However, a lack of information is cited as one of the problems. This could be eliminated by means of targeted education and information events. More serious problems such as lack of time and staff are not easily solved. Despite these hurdles, hypnosis techniques appear to be easy to incorporate into the anaesthetic procedure. However, time and staffing problems were cited as the biggest hurdles.

As shown by Lachkar et al. in 2022, the use of VR goggles is both effective in reducing anxiety during bronchoscopy under local anaesthesia and relatively easy to integrate into perioperative procedures [38]. They also show an anxiety-reducing effect on patients during other procedures under local anaesthesia [39] VR glasses can also be used for pain management of traumatological patients and thus contribute to the multimodal therapy concept [8]. In the interests of patient well-being, it is certainly expedient to continue to explore modern technical therapy options in anaesthesia. Regional anaesthesia seems to be particularly suitable for this purpose.

Uldal et al. have shown in their studies of gynaecological patients that they benefit from hypnosis procedures in the prenatal phase of obstetrics [40]. Hypnosis prior to surgery also proved to be useful in breast cancer surgery as fewer hypnotics and perioperative analgesics had to be used overall. Preoperative anxiety and postoperative complaints such as nausea were less pronounced when hypnosis was used [13, 41]. According to our survey, gynaecology and obstetrics are the specialist areas in Germany where hypnosis techniques are used very frequently.

According to the 2022 annual report of the Academy of Trauma Surgery, in 2021 there were 12.513 patients in Germany, Austria and Switzerland over the age of 70 who had sustained a femur fracture (including periprosthetic and peri-implant fractures) and therefore required surgical treatment [42]. Geriatric patients with fractures of the large tubular bones have an increased risk of postoperative complications, such as delirium. This is partly due to the use of substances that alter the vigilance and opioids as well as the patient’s own risk factors. As such substances are administered systemically during general anaesthesia procedures, it would be advantageous to use other procedures such as regional or local anaesthesia. However, premedication to reduce anxiety already plays a role in the development of delirium, so it would make sense to use a method that does not require medication to reduce stress and anxiety. According to our study hypnosis and relaxation techniques are used more frequently in trauma surgery and orthopaedics than in other specialties. This could be because preoperative anxiolytic medication is used more cautiously in older patients. For this reason, our questionnaire distinguished between children and adults, but not the exact age of the adults. A further survey would be needed to be sure of the number of geriatric patients that are treated with hypnosis.

Most hospitals using hypnosis treat both children and adults (82%). During a general anaesthetic, playing soothing words and suggestions to the patient via headphones has been shown to be effective in inducing hypnosis for pain, opioid use and on recovery [43, 44]. Various studies have also shown the positive effects of hypnosis on children in terms of heart rate, breathing rate, anxiety and therefore stress levels or the reduced need for painkillers [45, 46]. It is therefore surprising that only six out of 39 hospitals have stated that they use hypnosis in paediatric surgery. However, it is possible that in some hospitals children are also cared for by other specialties and are therefore not explicitly covered by paediatric surgery.

### Limitations

We suspect that the percentage of departments that actually use evidence-based hypnosis may be lower than recorded in the survey, as it is most likely that departments that do not use hypnosis did not participate in the survey. Due to the questionnaire we designed, there may be a social desirability bias, and it is possible that the respondents were more likely to select the perceived desirable answer of “yes”. Although positive images and suggestions may be used in the anaesthesia inductions, it is possible that they are not used with the specific background knowledge of a scientifically proven technique. This is supported by the fact that 8 out of 39 (21%) departments reported using hypnosis techniques without training [6] or with undefined in-house training [2]. However, there is evidence for the positive outcome of hypnotherapy when performed by certified practitioners. Moss and Willmarth showed this in their 2019 study [47].

Another limitation of the informative value of the data we collected is that we only interviewed clinic directors and not other doctors or health care workers. This means that there is no comprehensive picture of the opinions of all those involved in anaesthesia, particularly with regard to the desire for training and further education in hypnosis techniques.

## Conclusion

Hypnosis and relaxation techniques are only used to a limited extent in German hospitals with anaesthesia departments. The reasons for this may be a lack of knowledge about the techniques themselves, how they are performed and their proven effectiveness. It is obvious that a solution to this problem could be increased training in the field of hypnosis. There are also staffing and time difficulties in the perioperative process. When hypnosis or relaxation techniques are used, they are usually hypnotic trance according to Erickson, progressive muscle relaxation according to Jacobson, and suggestive words during or before induction of anaesthesia. More modern methods using VR glasses are also used. Given the generally good data available on the effectiveness of hypnosis and relaxation techniques more training and information programmes on this method should be established in Germany.

## Data Availability

Data and material are available on reasonable request. Inquiries can be sent to: christian.volberg@staff.uni-marburg.de.
